# Characteristics and Lethality of a Novel Recombinant Dermonecrotic Venom Phospholipase D from *Hemiscorpius lepturus*

**DOI:** 10.3390/toxins9030102

**Published:** 2017-03-13

**Authors:** Elham Torabi, Mahdi Behdani, Mohammad Hosseininejad Chafi, Reza Moazzami, Jean-Marc Sabatier, Vahid Khalaj, Delavar Shahbazzadeh, Kamran Pooshang Bagheri

**Affiliations:** 1 Venom and Biotherapeutics Molecules Lab., Medical Biotechnology Department, Biotechnology Research Center, Pasteur Institute of Iran, Tehran 13169-43551, Iran; e.torabi88@gmail.com (E.T.); behdani73042@yahoo.com (M.B.); hoseininejad62@gmail.com (M.H.C.); 2 Medical Biotechnology Department, Biotechnology Research Center, Pasteur Institute of Iran, Tehran 13169-43551, Iran; r.moazzami@gmail.com (R.M.); v_khalaj@yahoo.com (V.K.); 3 INSERM UMRs 1097, Aix-Marseille Université, Parc Scientifique et Technologique de Luminy, Marseille 13288, France; sabatier.jm1@libertysurf.fr

**Keywords:** *Hemiscorpius lepturus*, scorpion, phospholipase D, homology, brown spiders, Loxoscelidae family, lethality, dermonecrotic activity

## Abstract

*Hemoscorpius lepturus* is the most medically important scorpion in Iran. The clinical signs of *H. lepturus* envenomation are remarkably similar to those reported for brown spiders, including dermonecrosis, hematuria, renal failure and even death. The lethality and toxicity of brown spiders’ venom have been attributed to its phospholipase D activity. This study aims to identify a phospholipase D with possible lethality and dermonecrotic activity in *H. lepturus* venom. In this study, a cDNA library of the venom glands was generated by Illumina RNA sequencing. Phospholipase D (PLD) from *H. lepturus* was characterized according to its significant similarity with PLDs from brown spiders. The main chain designated as Hl-RecPLD1 (the first recombinant isoform of *H. lepturus* PLD) was cloned, expressed and purified. Sphingomyelinase, dermonecrotic and lethal activities were examined. Hl-PLD1 showed remarkable sequence similarity and structural homology with PLDs of brown spiders. The conformation of Hl-PLD1 was predicted as a “TIM beta/alpha-barrel”. The lethal dose 50 (LD_50_) and dermonecrotic activities of Hl-RecPLD1 were determined as 3.1 µg/mouse and 0.7 cm^2^ at 1 µg respectively. It is the first report indicating that a similar molecular evolutionary mechanism has occurred in both American brown spiders and this Iranian scorpion. In conclusion, Hl-RecPLD1 is a highly active phospholipase D, which would be considered as the lethal dermonecrotic toxin in *H. lepturus* venom.

## 1. Introduction

Scorpionism is one of the most important public health problems, involving 2.3 billion inhabitants in tropical, subtropical and temperate regions [1]. Scorpion stings have been frequently reported in Iran [2] with the most common incidence occurring in the Khuzestan province (541/100,000 people; 2.2% of the population) [3,4].

Among all scorpion species in Iran, there are six species that are medically important and responsible for scorpionism, including *H. lepturus*, *Odontobuthus doriae*, *Mesobuthus eupeus*, *Androctonus crassicauda*, *Buthotus saulcyi* and *Buthotus sach* [5]. The most medically important scorpion in Iran is *H. lepturus,* which belongs to Hemiscorpiidae family [6]. Excluding *H. lepturus*, almost all of the other venomous scorpion species in Iran belong to the large family of Buthidae [7].

Although only 15% of scorpion stings have been attributed to *H. lepturus,* it is the most lethal scorpion and accounted for 89% of deaths [8]. The mortality rate from *H. lepturus* envenomation is approximately 60 times higher than the other Iranian scorpions [8]. The most severe toxicity has been primarily reported in children [9]. Unlike other scorpions, the venom of *H. lepturus* is highly cytotoxic which could be the reason for its complicated clinical manifestations in envenomed people [10]. The sting of *H. lepturus* leads to various symptoms, including cutaneous reactions varying from undetectable small areas of erythema to large areas of dermonecrotic lesions at the sting site. In severe cases, the scorpion sting can trigger systemic effects characterized by persistent inflammation, vascular leakage, platelet aggregation, cardiovascular disease, severe hemolysis and hematuria, which can lead to renal failure [8,11,12,13]. These systemic disturbances may be attributable to some enzymatic components in the venom of *H. lepturus* [12]. Moreover, an increased level of liver enzymes (i.e., aspartate aminotransferase (AST), alanine aminotransferase (ALT) and alkaline phosphatase (ALP)) has also been reported as a potential consequence of this venom, indicating hepatocellular damage [10].

Our comprehensive literature review revealed that the major clinical signs of *H. lepturus* envenomation [8,11,12,13,14] are similar to those caused by *Loxosceles* genera (brown spiders) [15,16,17]. This observation may indicate the existence of major homologous toxins in both venoms. The dermonecrotic toxins are major molecules in the crude venom of brown spiders, being responsible for many clinical complications and deaths in humans. These dermonecrotic toxins were characterized as sphingomyelinase D (SMase D) in previous studies [18,19,20,21]. The toxins are able to hydrolyze sphingomyelin (SM), the major component of the outer leaflet of plasma membranes, into choline and ceramide 1-phosphate [18,19,20,21]. Sphingomyelinase D in brown spiders can act as a phospholipase D and its metabolites are important lipid mediators involved in several pathological and physiological processes [22,23,24,25,26]. It has been shown that different SMase D isoforms are simultaneously existed in the venom of a brown spider. These isoforms have various levels of sphingomyelinase, hemolytic, and lethal activities [19,21,26]. A similar SMase D molecule, designated as Heminecrolysin (HNc), has been partially sequenced and characterized as a dermonecrotic toxin in *H. lepturus* venom. However, its lethal activity has not been assessed yet [27]. 

According to our best of knowledge, no studies have been conducted to characterization of *H. lepturus* regarding the reason of venom lethality. Thus, it is necessitated to discover a responsible lethal toxin in *H. lepturus* venom. In the present study, we identified a novel recombinant phospholipase D responsible for the lethality and dermonecrotic activity of *H. lepturus* venom using both bioinformatic and functional assays.

## 2. Results

### 2.1. Selection and Characterization of the Candidate Open Reading Frame (ORF) and Main Chain

**Figure 1:**
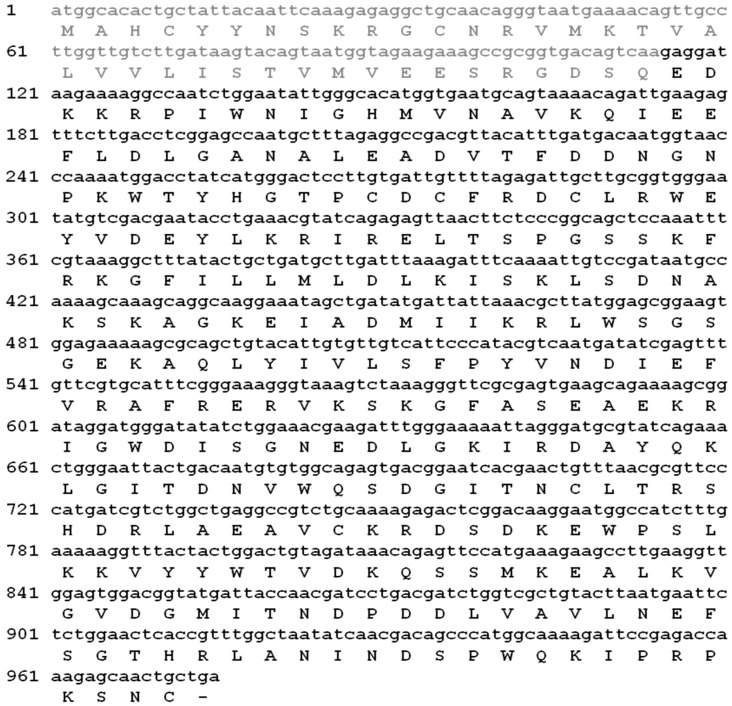
The putative amino acid sequence of *H. lepturus* PLD1. The mature main chain was determined as 286 amino acids and bolded as black.

Based on the results of homology analysis using protein-protein basic local alignment search tool (BLASTP), the best ORF with highest identity was selected. The candidate ORF and its main chain were determined as chains with lengths of 324 and 286 amino acids respectively (Figure 1). Hl-PLD1 demonstrated the highest sequence similarity with the isoforms of PLD from *L. intermedia* (identity 46%–48%). Other PLDs from Loxoscelidae were highly similar to Hl-PLD1 (identity 45%–49%).

Molecular weight, isoelectric point (pI) and Grand Average of hydropathicity (GRAVY) of the main chain were calculated as 32,720.20 Da, 7.77 and -0.58 respectively.

### 2.2. Homology Analysis of the Target Phospholipase D

**Figure 2:**
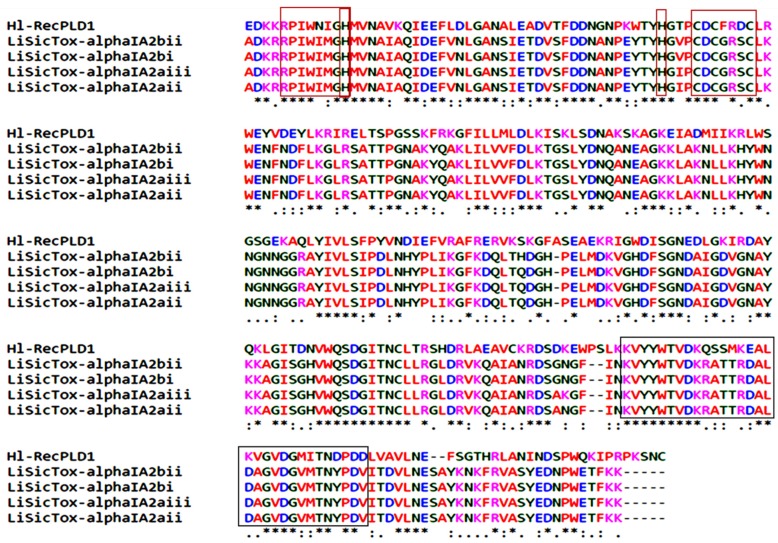
Sequence homology of Hl-PLD1 with PLD isoforms from *L. intermedia* by ClustalW analysis. The main chain of Hl-PLD1 was compared to its closest homologous proteins, PLDs from *L. intermedia*. As shown in the figure, *H. lepturus* and brown spiders have highly similar active sites and conserved regions (red boxes) in addition to catalytic site (black box).

ClustalW analyses showed that almost all of the Loxoscelidae PLDs (especially *L. intermedia*) were similar to Hl-PLD1 (Figure 2). A conserved motif at N-terminal region, “RPIWXXGH”, in *L. intermedia* PLDs was a suitable key point to use for prediction of the Hl-PLD1 main chain. According to homology comparison between the mature chain of Hl-PLD1 and *L. intermedia* PLDs, the “CXCXXXC” motif and two histidine residues (H12 and H48) were determined as a conserved region and active sites respectively. The catalytic site of Hl-PLD1 was determined based on the previous studies examining Loxosceles PLDs (Figure 2). 

### 2.3. Prediction of 3D Structure for Hl-PLD1

**Figure 3:**
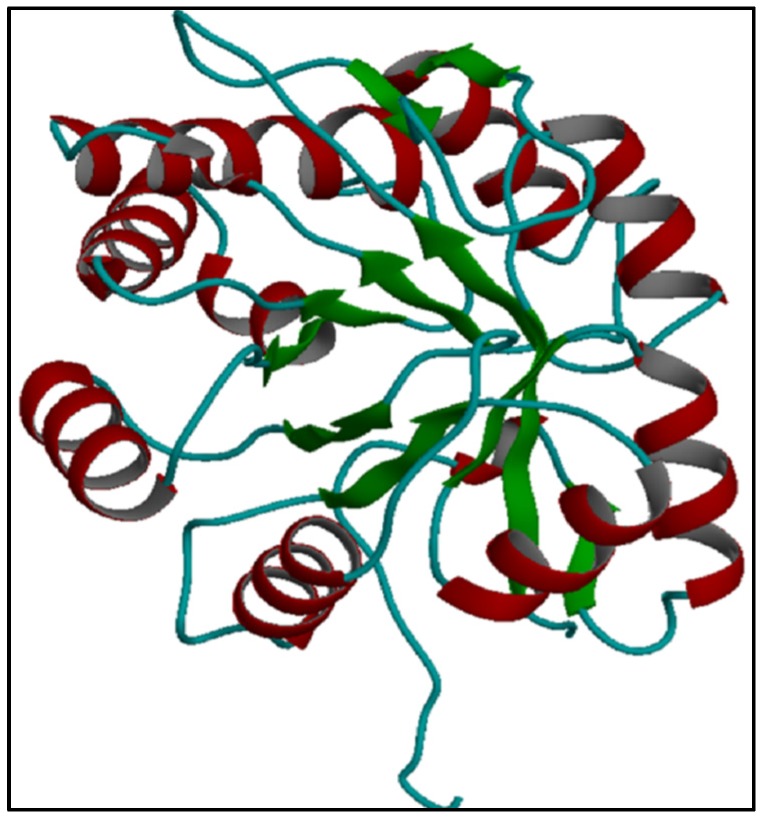
Prediction of the 3D structure of *H. lepturus* PLD1. The predicted conformation was determined as “TIM beta/alpha-barrel” in reference to its remarkable homology with *L. intermedia* PLD isoform, LiSicTox-alphaIA1bii.

The results indicate that the predicted confirmation contains several central parallel beta sheets and a number of peripheral alpha helices (Figure 3). The predicted structure was relatively similar to a phospholipase D isoform from *L. intermedia*, LiSicTox-alphalA1bii [28]. According to this significant similarity, Hl-PLD1 was classified as having a “TIM beta/alpha-barrel” structure. Beta sheets, alpha helices and coils were calculated by AntheProt 3D software as making up 17.1%, 35.3% and 47.6% respectively of the predicted structure. 

### 2.4. Structural Homology of Hl-PLD1 with Loxosceles Phospholipase D

**Figure 4:**
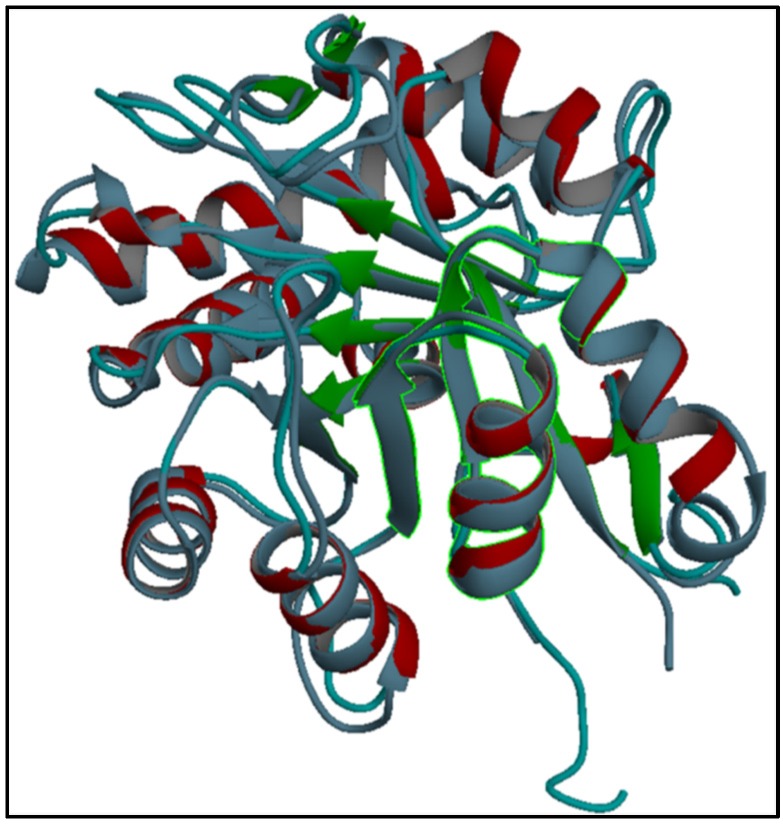
Structural alignment of the 3D structure of *H. lepturus* PLD1. Superimposition of the structure of LiSicTox-alphaIA1bii with the predicted structure of Hl-PLD1 showed that both toxins were significantly matched together. The catalytic sites of both PLDs were structurally the same (highlighted in green).

UCSF Chimera software performed a structural alignment of the predicted form of Hl-PLD1 with one of the previously determined PLD from *L. intermedia*, isoform LiSicTox-alphaIA1bii. The results clearly showed a significant structural homology between these isoforms (Figure 4). The structure of LiSicTox-alphaIA1bii was classified as “TIM beta/alpha-barrel” according to Coronado et al. in 2015 [28].

### 2.5. Verification of the Main Chain Sequence by Reverse Transcriptase-Polymerase Chain Reaction (RT-PCR) and DNA Sequencing

**Figure 5:**
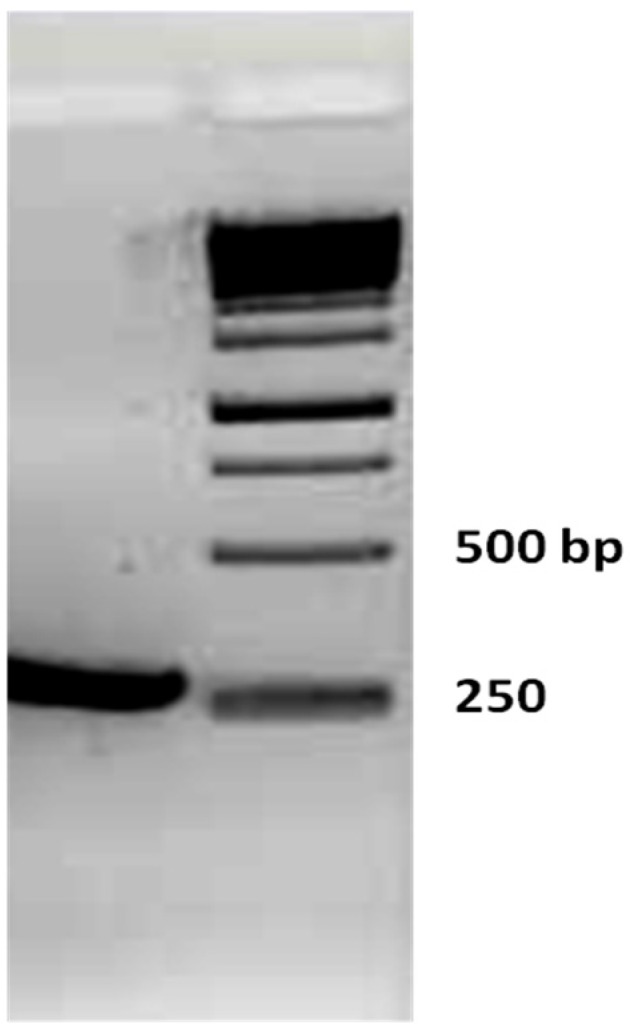
Gel electrophoresis of RT-PCR product. PCR product, negative control and DNA ladder are shown from left to right. A single pure DNA band at molecular weight of about 250 bp was seen, which primarily verified the accuracy of main chain by correct amplification of the desired internal fragment of Hl-PLD1 sequence.

The accuracy of the main chain sequence was confirmed by RT-PCR and DNA sequencing. A PCR product of approximately 250 bp confirmed correct amplification of the internal fragment corresponding to the Hl-PLD1 sequence (Figure 5). Further verification was achieved by sequencing of RT-PCR products.

### 2.6. Expression and Purification of the Recombinant Toxin

**Figure 6:**
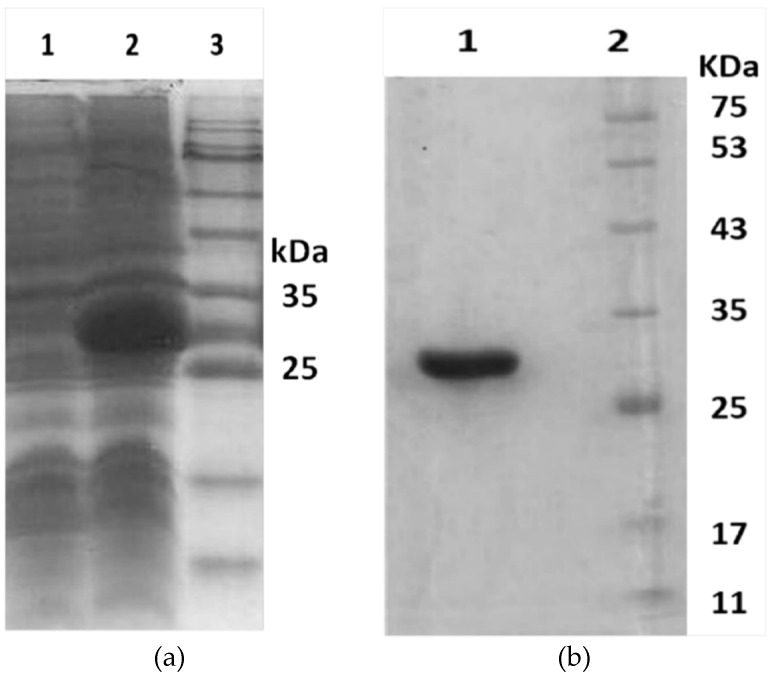
SDS-PAGE analysis of the expressed and purified Hl-RecPLD1. (**a**) The lanes 1, 2 and 3 showed uninduced and induced *E. coli* BL21 (DE3) and protein size marker (Cinaclon Co., Tehran, Iran) respectively; (**b**) The Lanes 1 and 2 showed purified eluted recombinant toxin and protein size marker (Cinaclon Co., Tehran, Iran).

The recombinant toxin herein was designated as Hl-RecPLD1. Figure 6a demonstrates the successful expression of H1-RecPLD1. Sodium Dodecyl Polyacrylamide Gel Electrophoresis (SDS-PAGE) showed a single purified protein at the expected weight of Hl-RecPLD1 (~32 KDa) (Figure 6b).

### 2.7. Immunoblotting of Purified Recombinant Toxin

**Figure 7:**
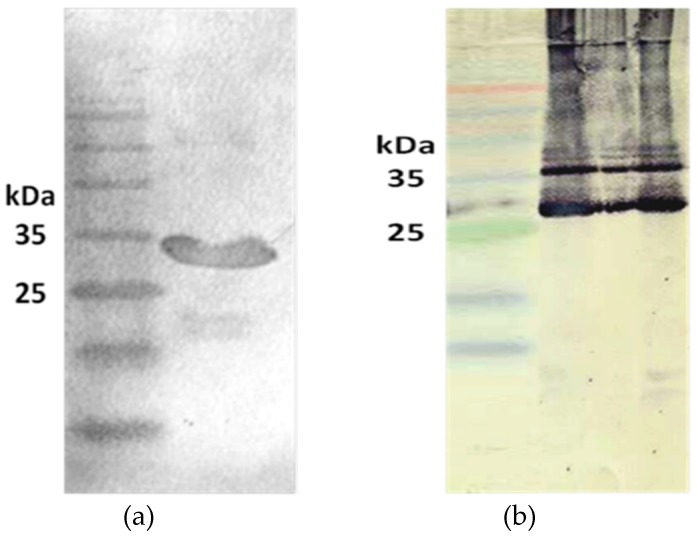
Immunoblotting of purified recombinant toxin and *H. lepturus* crude venom. (**a**) Horse anti-crude venom detected purified Hl-RecPLD1 at the molecular weight of about 32 kDa; (**b**) A confirmatory Western blot on the crude venom verified the existence of Hl-PLD1.

A horse was immunized with *H. lepturus* crude venom at intervals of 14 days over the course of 70 days. The highest titer of horse antibody was obtained in the 5th injection. Western blot analysis was performed for recombinant toxin using horse antisera. As shown in Figure 7, the recombinant phospholipase D, Hl-RecPLD1, was recognized by horse antibodies.

### 2.8. Sphingomyelinase Activity

**Figure 8:**
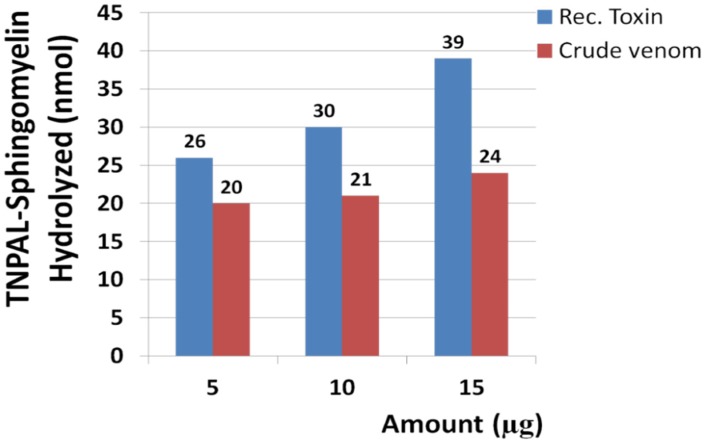
Sphingomyelinase activity of the recombinant toxin and crude venom. As shown, the sphingomyelinase activity of the recombinant toxin and crude venom increased in a dose-dependent manner. Sphingomyelinase activity of Hl-RecPLD1 was 1.4-fold higher than the crude venom (positive control).

The results showed that Hl-RecPLD1 at the amounts of 5, 10 and 15 µg was able to hydrolyze 26, 30 and 39 nmol of trinitrophenyl-aminolauryl-sphingomyelin (TNPAL-SM, Sigma Chemical Co., St Louis, MO, USA) respectively. At these same amounts, crude venom hydrolyzed 20, 21 and 24 nmol of the substrate respectively (Figure 8). Sphingomyelinase activity of Hl-RecPLD1 was 1.4-fold higher than the crude venom (positive control) at all examined amounts. Increased amounts of recombinant toxin led to increased sphingomyelinase activity.

### 2.9. Dermonecrotic Effect of Hl-RecPLD1 on Rabbit Skin

**Figure 9:**
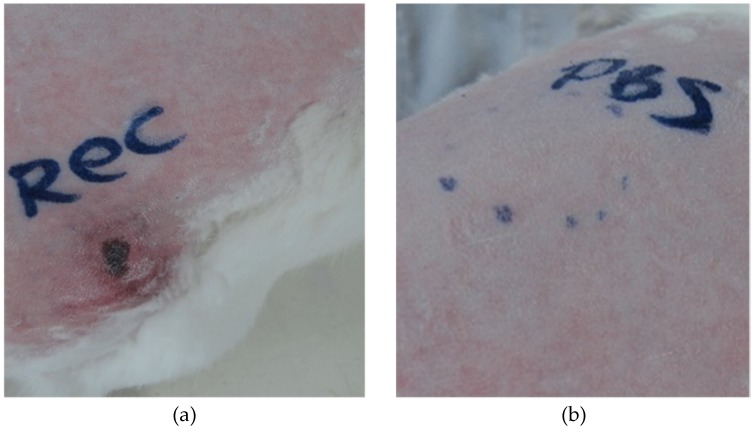
Macroscopic visualization of necrotic response to the recombinant toxin. (**a**) Hl-RecPLD1 (1 μg) showed a clear dermonecrotic activity on rabbit skin. The lesions were observed during 6, 24, 48 and 72 h following injection and the subsequent necrotic area reached 0.7 cm^2^; (**b**) Phosphate buffered saline (PBS, 1x) was used as a negative control.

To determine the dermonecrotic activity of Hl-RecPLD1, 1 µg of recombinant toxin was intradermally injected into rabbit skin. The lesion area was checked and measured. The necrosis area reached approximately 0.7 cm^2^ after 72 h (Figure 9a,b).

### 2.10. Mouse Mortality of Hl-RecPLD1

The mortality induced by recombinant toxin in the mice was documented for 4 days after intraperitoneal injections. The LD_50_ and lethal dose 100 (LD_100_) of the recombinant toxin were determined as 3.1 and 3.7 µg respectively for each mouse. 

### 2.11. Hemolytic Activity

No hemolytic activity was detected with injections of up to 50 µg with or without human serum.

## 3. Discussion

*H. lepturus* is one of the most dangerous scorpions in the Khuzestan province of Iran. *H. lepturus* venom is very toxic and some of its peptide and protein components have been completely or partially characterized, such as Hemitoxin [29], Hemicalcin [6], Heminecrolysin [27] and Hemilipin [30].

The present study is the first report on high sequence similarity between a cytotoxic scorpion in Asia and brown spiders (*L. intermedia*) from America. This level of sequence similarity was a significant landmark to the discovery of *H. lepturus* PLD and also for determination of the main chain, active and catalytic sites of *H. lepturus* PLD.

Two evolutionary signatures, two active sites and a region of 31 amino acids corresponding to phospholipase D activity were determined according to the BLASTP and ClustalW analyses between Hl-PLD1 and isoforms of PLDs from *L. intermedia*. Prediction of the three-dimensional structure of Hl-PLD1 produced a “TIM beta/alpha-barrel” that had significant structural homology with the predetermined structure of *L. intermedia* LiSicTox-alphaIA1bii isoform [28]. It is the first report suggesting that similar molecular evolutionary mechanisms happened in two different organisms existing in distinct geographical areas, Iran and America.

In reference to the significant homology of Hl-PLD1 with dermonecrotic toxins of *L. intermedia* (i.e., amino acid sequence, 3D structure, active sites and catalytic site), we proposed that Hl-PLD1 and *L. intermedia* PLDs have similar activity, toxicity and lethality.

According to bioinformatic analyses, the main chain for Hl-PLD1 was determined and the sequence was cloned in pET-22b as a recombinant form of Hl-PLD1 (Hl-RecPLD1). Hl-RecPLD1 was expressed and purified as a ~32 kDa protein. The horse polyclonal antibody against the crude venom, developed in this study, was able to recognize Hl-RecPLD1. The existence of Hl-PLD1 in *H. lepturus* crude venom was confirmed by employing Western blotting.

Hl-RecPLD1 is a highly active enzyme that was able to hydrolyze the TNPAL-SM substrate in the sphingomyelinase assay. It was able to hydrolyze the substrate around 40- and 1.4-fold greater than the HNc reported by Borchani et al. [27] and the crude venom used in this study, respectively. Similarly, in *L. intermedia* venom, some of its recombinant PLD isoforms (i.e., LiRecDT1 and LiRecDT4) are more active than the *L. intermedia* crude venom [19]. A comparison between the amount of the substrate hydrolyzed by HNc [27] and the *H. lepturus* crude venom in our study showed that the sphingomyelinase activity of HNc is less active than the crude venom. In this regard, HNc is similar to LiRecDT5 in the *L. intermedia* venom in that it has the least sphingomyelinase activity compared to LiRecDT1, LiRecDT4 and the *L. intermedia* crude venom [19]. Kalapothakis et al. described in 2007 this variation in sphingomyelinase activity of some PLD isoforms derived from *L. intermedia* [31]. 

A small amount of Hl-RecPLD1 at LD_50_ = 3.1 µg was able to induce death in mice. This notably small quantity of the toxin cannot hydrolyze all of the sphingomyelin molecules in all cell surfaces. It is rationally proposed that the sphingomyelinase activity of Hl-RecPLD1 can act as a trigger for lethality. This hypothesis originated from previous studies, which provided evidence that metabolites of sphingomyelin hydrolyzed by PLDs in brown spiders are responsible for pathological events after envenomation [21,22,23,24,25,26]. In our study, a slow trend of mouse mortality was observed during 96 h after injection. This observation pointed out the slow progression of toxicity effects. This approach could be very important in the study of toxicity in animal models as complications have been known to occur slowly in envenomed patients [13].

Noxious responses, such as a lesion with swelling, erythema and necrosis, were observed upon injection of the recombinant toxin into rabbit skin. These signs are a result of the sphingomyelinase activity of Hl-RecPLD1. It is hypothesized that the enzymatic activity of Hl-RecPLD1 caused tissue degradation, subsequent inflammation and necrosis. This dermonecrotic activity has been shown in HNc and the recombinant isoforms of dermonecrotic toxins from *L. intermedia* as well [19,21,26,27]. Based on our study, *H. lepturus* PLD1 is one of the dermonecrotic toxins existing in the venom of the Iranian scorpion, *H. lepturus*.

Hl-RecPLD1 did not show any hemolytic activity up to 50 µg. This issue confirmed that Hl-PLD1 differs from HNc and various isoforms of recombinant PLDs from *L. intermedia* [27,32]. To control the effect of the complement system on hemolysis, fresh human serum was incubated with human RBCs in the presence of different amounts of Hl-RecPLD1. No hemolysis was seen again whereas in another study, Borchani et al. described the positive effect of the complement system on hemolysis of native HNc [27]. It can be concluded that the mortality of the mice was not attributed to hemolysis. 

The above-mentioned complications have been documented in human victims and currently, the only effective therapy for scorpionism is F(ab)’_2_ or Fab antibody fragments that are produced in immunized horses [33]. This kind of immunotherapy has serious limitations in terms of specificity [34]. In this case, inhibition of *H. lepturus* PLD1 activity might be a suitable suggestion by employing monospecific antibodies or nanobodies against recombinant PLD1 to prevent lethality or complications after envenomation.

Due to the painless nature of *H. lepturus* envenomation [11] and the slow development of subsequent symptoms, the envenomed patients often are not admitted to hospital. These conditions provide enough time for the toxins to exert their toxic activities, after which subsequent complications or death will happen. These concerns and high incidence rate of *H. lepturus* envenomation in the southwest of Iran [3,4,8] encourage us to develop a vaccine. As Hl-RecPLD1 is a major lethal toxin, it might be an appropriate candidate molecule for immunization to prevent the noxious complications of envenomation. 

As indicated by Laustsen et al. [35] in 2016, the discovery of new toxins from scorpions and spiders are of remarkable value for biotechnological applications and the development of new drugs. According to the sphingomyelinase activity of Hl-RecPLD1, it could be of interest in anticancer studies. This suggestion arises from the fact that glycerophospholipids and sphingolipids are involved in various pathophysiological processes, like G protein-coupled receptors (GPCRs) and kinase cascades [22,36,37]. As Hl-RecPLD1 can degrade sphingomyelin molecules on the cell membrane, it is proposed that it may interfere in signaling pathways in cancerous cells.

In conclusion, Hl-RecPLD1 was characterized as a highly active phospholipase D from the venom of the Iranian scorpion, *H. lepturus*. According to results obtained from study of the recombinant PLD1, it appears that Hl-PLD1 in *H. lepturus* venom could be considered as a novel lethal, dermonecrotic toxin. Taken together, these results will provide insight into scorpionism in addition to contributing to further pathological evaluations and biotechnological applications of this recombinant toxin.

## 4. Materials and Methods

### 4.1. Specimen Collection

**Figure 10:**
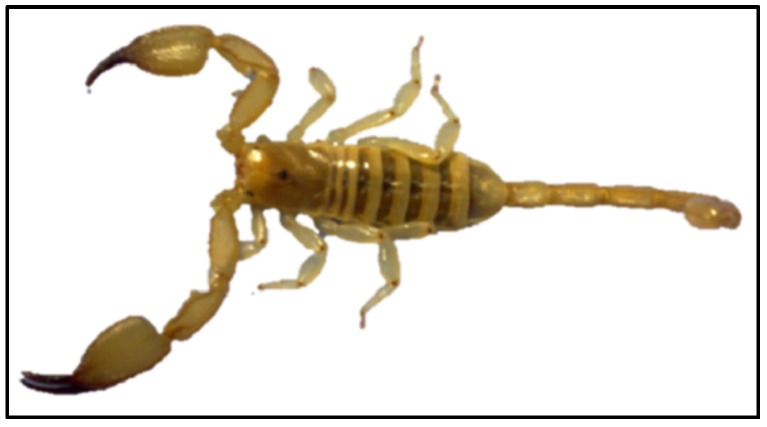
A photographic illustration of *Hemiscorpius lepturus*. The yellow brown body and dark brown chelicerae are unique characteristics used for identification of this species.

**Figure 11:**
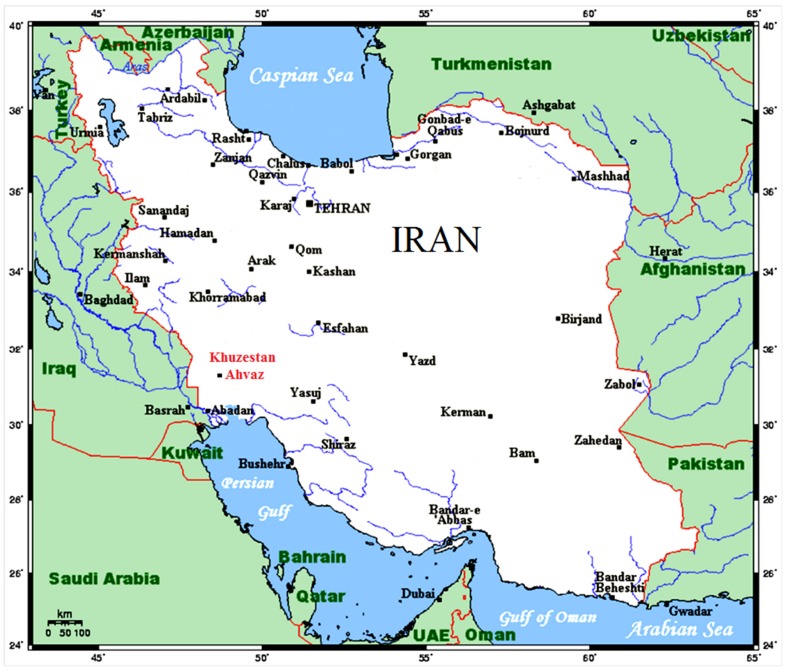
Specimen collection area. Ahvaz City in the southwest of Iran (31°00′ N 49°00′ E).

*Hemiscorpius lepturus* is usually less than 9 cm in length and can be easily distinguished by its unique bead-shaped jointed tail, dark brown chelicerae and yellow–brown body (Figure 10). One hundred *H. lepturus* samples were collected manually from the suburban area of Ahvaz city in the Khuzestan province of Iran (31°00′ N 49°00′ E), with the specimens then being transferred to the laboratory (Figure 11).

### 4.2. cDNA Library Construction and Sequencing

The venom of *H. lepturus* scorpions were collected by the milking method [38] and two days later, the venom glands were cut and kept frozen in liquid nitrogen. The glands were cut into small pieces and grounded in liquid nitrogen to obtain a fine powder. The powder was re-suspended in Trizol (Invitrogen Co., Carlsbad, CA, USA) and centrifuged at 12,470 g. The quality of the extracted RNA was checked by electrophoresis on 1.5% agarose gel at 70 V for 40 min using Tris Acetate EDTA (TAE, 1x) buffer. The extracted RNA was transferred to an external facility (Source BioScience Co., Nottingham, UK).

### 4.3. Bioinformatics Analyses

#### 4.3.1. Data Mining and ORF Determination

Our desired keywords, such as phospholipase D, glycerophosphoryl diester phosphodiesterase and sphingomyelinase, were traced in our Hemiscorpius databank provided by the company. The DNA sequences representing phospholipase annotation were extracted and translated to ORF by both expasy translate [39] and ORF finder servers [40]. Several phospholipase D sequences were identified in *H. lepturus* venom, indicating the possible existence of phospholipase D isoforms.

#### 4.3.2. Homology Analysis of the Target Phospholipase D and Determination of Main Chain

Among our phospholipase D ORFs, the best one was selected for characterization based on its highest sequence similarity with phospholipase D from the *Loxosceles* species. Sequence similarity of the candidate ORF was determined against non-redundant (nr) and reference sequences by BLASTP analysis. For an accurate determination of the main chain, the identified ORF for *H. lepturus* PLD was aligned with other members of the Loxoscelidae family, taxid 6919, by ClustalW in MEGA6. Sequence similarity of the catalytic sites of *Loxosceles* PLDs was evaluated against *H. lepturus* PLD sequence and subsequently, the catalytic site was predicted using ClustalW in MEGA6 software and also on the MUSCLE (MUltiple Sequence Comparison by Log-Expectation) server [41,42]. All similar protein amino acid sequences were estimated for several key points including conserved motifs, active sites and catalytic site in the Uniprot server [43], which were characterized in reference to *L. intermedia* PLD sequences [19,21]. Based on the obtained results, the main chain of *H. lepturus* PLD was ultimately predicted and designated as Hl-PLD1 (the first isoform of *H. lepturus* PLD).

The molecular weight, pI and also the grand average of hydropathicity of the main chain were calculated by the Protparam server [44].

#### 4.3.3. Prediction of Hl-PLD1 Conformation

The three-dimensional structure of Hl-PLD1 was predicted in the I-TASSER server (Iterative Threading ASSEmbly Refinement) in Michigan University [45]. I-TASSER is a hierarchical approach to protein structure and function prediction. Structural templates are first identified from the PDB by the multiple threading approach designated Local Meta-Threading-Server (LOMETS). Full-length atomic models are then constructed by simulations of iterative template fragment assembly. I-TASSER outputs include at least five models. Model one was selected due to having the lowest Root Mean Square Deviation (RMSD). It means that the predicted structure has the lowest error during structural modeling [46]. The predicted structure will be used for comparison of the structural homology between Hl-PLD and the predetermined structure of PLDs derived from *L. intermedia*. 

#### 4.3.4. Structural Homology of Hl-PLD1 with *Loxosceles* Phospholipase D

The aim of this assay was to predict the correlation between activity and conformation, especially in the catalytic site. It means that similarity is not limited to the sequence. The predicted conformations of Hl-PLD1 and its catalytic site were superimposed on the crystal structure of one of the PLD isoform from *L. intermedia*, LiSicTox-alphaIA1bii. Structural alignments were performed and visualized using the UCSF Chimera software (ver. 1.11.2, University of California, San Francisco, CA, USA). Pairwise alignment was checked by the “MultAlignViewer” tool in Chimera to show the percent of structural identity between Hl-PLD1 and LiSicTox-alphaIA1bii isoform [47]. 

### 4.4. Verification of Determined Sequence by RT-PCR

#### 4.4.1. RT-PCR

To confirm the accuracy of the sequence obtained by Illumina technique, RT-PCR was performed using our designed primers. Briefly, four glands of *H. lepturus* were pulverized and grounded into a fine powder in the presence of liquid nitrogen. Total RNA was extracted by the RNX plus reagent (CinaClone, Tehran, Iran) and used as a template. Gene-specific primers were designed against the internal region of the ORF using “Gene Runner” and Oligo Analyser software programs. The first strand cDNA was synthesized using oligo (TTTTTTTTTTTTTTTTTT)/(dT)_18_ primer employing Reverse Transcriptase. All the RT-PCR and PCR reagents were purchased from Thermo Fisher Scientific Co., Waltham, MA, USA. 

The RT-PCR was performed in a final volume of 20 μL, containing 240 ng RNA (4 μL), 2 μL of oligo (dT)_18_ primer (10 pm), 2 μL dNTP (10 mM), 5x RT-PCR buffer (4 μL), 1 μL Moloney Murine Leukemia Virus Reverse Transcriptase (200 U/μL M-MLV RT), 0.5 μL RNase inhibitor (Ribolock^®^, Thermo Fisher Scientific Co., Waltham, MA, USA., 40 U/μL) and 6.5 μL DEPC treated water. The mixture was initially heated at 72 °C for 10 min, followed by 42 °C for one hour and then chilled on ice until cDNA synthesis. The cDNA was amplified using the forward (5′-TGTCTTGATAAGTACAGTAATGGTAG-3′) and reverse primers (5′-GTGGGAATATGTCGACGAATACCTG-3′), employing Taq DNA polymerase. 

PCR was carried out in a reaction volume of 25 μL, containing 3 μL of the synthesized cDNA, 2.5 μL 10X PCR buffer, 1 μL of each primer (10 pmol), 0.2 μL Taq DNA polymerase (5 U/μL), 0.5 μL MgCl_2_ (1.5 mM), 0.5 μL dNTPs (10 mM) and 16.3 μL DEPC treated water. The procedure for PCR began with denaturation at 94 °C for 10 min, followed by 40 cycles of PCR steps including denaturation at 94 °C for 45 s, annealing at 55 °C for 45 s, and extension at 72 °C for 1 min. Finally, a further extension was performed at 72 °C for 10 min.

#### 4.4.2. Sequencing of RT-PCR Products

The RT-PCR product was electrophoresed on 2% gel agarose at 70 V for 45 min using TAE buffer (1x). The expected PCR product was purified by a gel extraction kit according to manufacturer instructions (Qiagen Co., Hilden, Germany) and sequenced in both directions by the Source Bioscience Company (Nottingham, UK).

The sequencing results were quality-controlled by Chromas software (ver. 2.4.3, Technelysium, South Brisbane, Australia). The similarity of the obtained sequence was compared to the desired sequence in our Hemiscorpius databank using the ClustalW tool in MEGA6 software. 

### 4.5. Cloning and Expression of Recombinant Toxin

The gene (GenBank accession number: KY287766) was codon–optimized for the *E. coli* strain K12. The gene designated as Hl-RecPLD1, with a 6x His-Tag at the C-terminus flanked by NdeI and XhoI, was synthesized by Generay Biotech Co. (Hong Kong, China) and inserted into pET-22b expression vector. 

The recombinant construct was transformed into *E. coli* BL21 (DE3) cells [48]. *E. coli* BL21 (DE3) cells were cultured in Luria-Bertani broth (LB) overnight. The cells were harvested at 3615 g for 5 min at room temperature and re-suspended in 100 µL cold CaCl_2_ (0.1 M) before being incubated on ice for 20 min. The cells were centrifuged and the pellet was washed two times in 100 µL cold CaCl_2_ (0.1 M) by centrifugation at 3615 g for 5 min. Subsequently, the pellet was re-suspended in 100 µL cold CaCl_2_ (0.1 M). The pET-22b expression vector (1 µg) was then added to the suspension and heated at 42 °C for 90 s followed by 2 min incubation on ice. Finally, 1 mL of LB broth was added to the suspension and incubated at 37 °C for one hour. The cells were centrifuged again at 3615 g for 5 min and re-suspended in 100 µL fresh LB broth. Fifty microliters of the transformants were plated on LB agar by ampicillin (100 µg/mL) overnight.

A single colony of the transformants was inoculated into LB medium containing ampicillin (100 µg/mL) overnight. Fifty microliters were then sub-cultured in fresh LB broth containing 100 µg/mL ampicillin (5 mL) to reach an optical density at 600 nm wavelength (OD_600_) of 0.6. Induction was performed using 0.1 mM isopropyl β-d-thiogalactoside (IPTG, Thermo Fisher Scientific Co., Waltham, MA, USA) and the gene was expressed for 3.5 h at 30 °C in a shaker incubator at 170 RPM. The cells were harvested by centrifugation (10,397 g, 10 min, 4 °C) (Sigma 3-18K, Osterode am Harz, Germany), and frozen at −20 °C.

### 4.6. Purification of Recombinant Toxin

Host cells were suspended in 15 mL of lysis buffer (50 mM sodium phosphate buffer, 300 mM NaCl and 10 mM Imidazole with a pH of 8.0) and disrupted through sonication (Hielscher Co., Teltow, Germany) at 30% amplitude and cycled 30 s for 4.5 min. The cell lysate was centrifuged at 10,397 g for 10 min at 4 °C before being filtered through a membrane with 0.22 µm porosity.

The Ni-NTA agarose (Qiagen Co., Hilden, Germany) column was washed and equilibrated with washing buffer (50 mM sodium phosphate buffer, 300 mM NaCl and 20 mM imidazole at a pH of 8.0). The suspension was loaded onto the column and was washed by washing buffer as mentioned above to remove the impurities. The recombinant proteins were eluted with 10 ml of elution buffer (50 mM sodium phosphate buffer, 300 mM NaCl and 250 mM imidazole at a pH of 8.0). The eluted fraction was collected and dialyzed against PBS (1x). To check the purity of the eluted Hl-RecPLD1, the collected fraction was subjected to SDS-PAGE, which was performed according to the Laemmli method [49] with some modification. The purified recombinant toxin was loaded onto a 15% polyacrylamide gel and electrophoresed at initially 15 mA for 30 min, followed by 25 mA for 2 h. Protein concentrations were determined using the BCA Protein Assay Kit according to manufacturer instructions (iNtRON Biotechnology Co., Seoul, Korea).

### 4.7. Western Blotting

To perform this step with specific antibodies, monovalent antisera were prepared as outlined below.

#### 4.7.1. Preparation of Horse Antisera against *H. lepturus* Venom

As the available industrial antiserum in Iran is polyvalent against the six medically important scorpions, monovalent horse antiserum against the venom of *H. lepturus* was produced as detailed below to avoid cross-reactions in Western blot analysis. 

#### 4.7.2. Horse Immunization with Crude Venom

To produce horse antibody against the crude venom of *H. lepturus*, immunization was performed in Iranian Turkmen horses collected from the northeastern area of Iran (37°15′ N 55°10′ E). At first, 100 µg was injected intradermally with Complete Freund’s Adjuvant (CFA, Sigma Co., St. Louis, MO, USA) into the cervical region based on the recommendation of the World Health Organization (WHO) [50]. After this, four increasing amounts of venom from 200 to 500 µg in rising steps of 100 µg were injected with Incomplete Freund’s Adjuvant (IFA, Sigma Co., St. Louis, MO, USA). The interval duration between injections was 14 days. 5 mL of serum was collected before each injection and 14 days after the final injection. This was kept frozen at −20 °C until used. 

#### 4.7.3. Antibody Assays 

To confirm the antibody production against the injected venom, ELISA was performed as outlined below. Briefly, 1 µg of the crude venom was suspended in 100 mM carbonate-bicarbonate buffer (pH 9.6) and was coated in a 96-well microplate (Nunc, Sigma Co., St. Louis, MO, USA) overnight at room temperature. After washing with PBS-Tween 20 (0.05%), the blocking step was performed with BSA (2%) for one hour at room temperature. Horse serum (100 µL) was serially diluted with PBS, added to each well and incubated at 37 °C for one hour. After three washing steps, HRP-labeled anti-horse antibody (Sigma Co., St. Louis, MO, USA) was added to each well, incubated at 37 °C for one hour, and washed as before. 3,3′,5,5′-Tetramethylbenzidine (TMB, Pishtaz Teb Zaman Diagnostics, Tehran, Iran) was added to each well, incubated for 15 min at room temperature in a dark room, and then the reaction was stopped with sulfuric acid (2 M). Optical density (OD) was finally measured at 450 nm. 

#### 4.7.4. Western Blotting 

Identification of the purified Hl-RecPLD1 was verified by employing Western blotting by horse anti-crude venom. The collected fraction from Ni-NTA column was visualized by SDS-PAGE as detailed above. For immunoblotting of the recombinant protein and crude venom, the samples were electrically transferred to nitrocellulose membranes using the semi-dry technique at 14 V for 45 min (Bio-Rad Co., Hercules, CA, USA). The membrane was blocked using skimmed milk (4%) at room temperature for one hour. Hyperimmune horse antiserum was used as the primary antibody, which was produced earlier at the step where horses were immunized. The primary antibody was incubated on the membrane at a titer of 1/1000 for one hour at room temperature. The washing step was done by PBS-tween20 for three times. HRP-labeled anti-horse antibody (Sigma Co., St. Louis, MO, USA) at the titer of 1/3000 was incubated for one hour at room temperature. Washing was performed as described above. The protein bands were visualized for the recombinant protein and the crude venom in separate membranes after color development with 3,3′-Diaminobenzidine tetrahydrochloride (DAB, Sigma, St. Louis, MO, USA) and 4-Chloro-1-Naphthol (4-CN, Sigma, St. Louis, MO, USA).

### 4.8. Sphingomyelinase Activity Assay for Hl-RecPLD1

Sphingomyelinase activity of the purified recombinant protein was assessed by measuring the enzyme-catalyzed hydrolysis of a sphingomyelin analog, TNPAL-SM, according to Borchani et al. in 2011 [27]. The increasing amounts of recombinant toxin and crude venom, including 5, 10 and 15 µg, were incubated with an incubation buffer (190 µL) containing 250 mM Tris–HCl, 20 mM MgCl_2_, 0.1% Triton X-100, and 60 nmol of TNPAL-SM at a pH of 7.4. The reaction mixtures were gently shaken for 2 h at 37 °C. Subsequently, the reactions were stopped by adding 375 µL of isopropanol/heptane/H2SO_4_ (40:10:1 *v*/*v*). 200 µL of both heptane and water were then added per sample. The tubes were centrifuged at 664 g for 5 min to separate the resulting two phases. The upper phase was transferred to a 96-well microplate and OD was read at 410 nm. 

The *H. lepturus* crude venom and PBS (1x) were used as positive and negative controls respectively. The sphingomyelin hydrolysis was expressed as the quantity of TNPAL-SM (as nmol) hydrolyzed per mg of toxin (1 nmol of hydrolyzed TNPAL-SM corresponds to 0.023 absorbance units) [27].

### 4.9. Animal Model Studies

Adult male BALB/c mice (25 g) and female New Zealand Albino rabbits (2–3 kg) were purchased from the Pasteur Institute of Iran. Upon arrival, the animals were allowed to adapt for a week before taking part in the experiment. The dark and light duration cycled every 12 h. The room temperature was 22 ± 1 °C and the relative humidity adjusted at 50% ± 5%. The animals were given a standard pellet diet and fresh clean tap water. All experiments involving animals were performed in accordance with the ethical committee on research animal care agreement in Pasteur Institute of Iran (approval number: IR.PII.REC.1394.86). All the tests were done in triplicate.

#### 4.9.1. Lethal Activity of Purified Hl-RecPLD1 in Mice

Mouse mortality studies were performed on BALB/c mice (20–30 g) using Spearman-Karber’s method [51]. Ascending amounts of the recombinant toxin, including 2.6, 3.1, 3.7, 4.5, 5.4, and 6.48 µg were prepared in 100 µL sterile PBS (1x) and injected intraperitoneally into each group (four mice/group). Sterile PBS (1x) and crude venom were used as negative and positive controls respectively. The mice were observed for 16, 20, 24, 48, 72 and 96 h after injection and LD_50_ was calculated based on the following formula:

$$log\;LD50=logX100-\left[\left(\frac{logfd}{n}\right)\;\times \left(\frac{\sum^{\text{​}}T}{n}\right)\right]\;$$

#### 4.9.2. Dermonecrotic Activity of Hl-RecPLD1

Purified recombinant toxin (1 µg) was prepared in 100 µL sterile PBS and injected intradermally into the back area of the rabbit skin. After 4, 8, 16, 24, 40, 48, 72 and 96 h, the injected area was evaluated regarding inflammation, erythema and necrosis. The area of inflammation or necrosis was measured in square centimeters. Sterile PBS (1x) and crude venom were injected intradermally as negative and positive controls respectively. 

### 4.10. Hemolytic Activity

This assay was performed as described in literature [52], with some modifications. Fresh human blood was obtained from a healthy donor and washed 3 times with PBS (1x). Serial dilutions of recombinant protein (from 50 to 0.39 µg) were prepared in 100 µL PBS (1x) and 100 µL of washed RBCs suspension (2%) was added to each well of a 96-well microplate (Nunc, Sigma Co., St. Louis, MO, USA). 

To evaluate the effect of serum on hemolysis, in a separate series of assays, serial dilutions of recombinant protein (from 50 to 0.39 µg) were prepared in 100 µL human serum. The washed RBCs (2%) were added to each well as detailed above. The microplate was incubated at 37 °C for 2 h and centrifuged at 664 g for 10 min. The supernatant was transferred to a new plate and OD was read at 540 nm in a microplate spectrophotometer (EPOCH, BioTek Co., Winooski, VT, USA). Triton X-100 (2%) and PBS buffer (1x) were used as positive and negative controls respectively. The degree of hemolysis was determined using the below formula.

$$\frac{\mathrm{OD}\;\mathrm{sample}\;-\;\mathrm{OD}\;\mathrm{negative}\;\mathrm{control}}{\mathrm{OD}\;\mathrm{positive}\;\mathrm{control}\;-\;\mathrm{OD}\;\mathrm{negative}\;\mathrm{control}}\times 100$$
